# Assessment of Skin Maturity by LED Light at Birth and Its Association With Lung Maturity: Clinical Trial Secondary Outcomes

**DOI:** 10.2196/52468

**Published:** 2023-12-25

**Authors:** Gabriela Silveira Neves, Zilma Silveira Nogueira Reis, Roberta Romanelli, James Batchelor

**Affiliations:** 1 Faculty of Medicine Universidade Federal de Minas Gerais Belo Horizonte Brazil; 2 Faculty of Medicine University of Southampton Southampton United Kingdom

**Keywords:** newborn infant, prematurity, neonatal respiratory distress syndrome, skin physiological phenomena, photometer, gestational age

## Abstract

**Background:**

Clinicians face barriers when assessing lung maturity at birth due to global inequalities. Still, strategies for testing based solely on gestational age to predict the likelihood of respiratory distress syndrome (RDS) do not offer a comprehensive approach to addressing the challenge of uncertain outcomes. We hypothesize that a noninvasive assessment of skin maturity may indicate lung maturity.

**Objective:**

This study aimed to assess the association between a newborn’s skin maturity and RDS occurrence.

**Methods:**

We conducted a case-control nested in a prospective cohort study, a secondary endpoint of a multicenter clinical trial. The study was carried out in 5 Brazilian urban reference centers for highly complex perinatal care. Of 781 newborns from the cohort study, 640 were selected for the case-control analysis. Newborns with RDS formed the case group and newborns without RDS were the controls. All newborns with other diseases exhibiting respiratory manifestations were excluded. Skin maturity was assessed from the newborn's skin over the sole by an optical device that acquired a reflection signal through an LED sensor. The device, previously validated, measured and recorded skin reflectance. Clinical data related to respiratory outcomes were gathered from medical records during the 72-hour follow-up of the newborn, or until discharge or death, whichever occurred first. The main outcome measure was the association between skin reflectance and RDS using univariate and multivariate binary logistic regression. Additionally, we assessed the connection between skin reflectance and factors such as neonatal intensive care unit (NICU) admission and the need for ventilatory support.

**Results:**

Out of 604 newborns, 470 (73.4%) were from the RDS group and 170 (26.6%) were from the control group. According to comparisons between the groups, newborns with RDS had a younger gestational age (31.6 vs 39.1 weeks, *P*<.001) and birth weight (1491 vs 3121 grams, *P*<.001) than controls. Skin reflectance was associated with RDS (odds ratio [OR] 0.982, 95% CI 0.979-0.985, *R*^2^=0.632, *P<*.001). This relationship remained significant when adjusted by the cofactors antenatal corticosteroid and birth weight (OR 0.994, 95% CI 0.990-0.998, *R*^2^=0.843, *P<*.001). Secondary outcomes also showed differences in skin reflectance. The mean difference was 0.219 (95% CI 0.200-0.238) between newborns that required ventilatory support versus those that did not and 0.223 (95% CI 0.205-0.241) between newborns that required NICU admission versus those that did not. Skin reflectance was associated with ventilatory support (OR 0.996, 95% CI 0.992-0.999, *R*^2^=0.814, *P*=.01) and with NICU admission (OR 0.994, 95% CI 0.990-0.998, *R*^2^=0.867, *P*=.004).

**Conclusions:**

Our findings present a potential marker of lung immaturity at birth using the indirect method of skin assessment. Using the RDS clinical condition and a medical device, this study demonstrated the synchrony between lung and skin maturity.

**Trial Registration:**

Registro Brasileiro de Ensaios Clínicos (ReBEC) RBR-3f5bm5; https://tinyurl.com/9fb7zrdb

**International Registered Report Identifier (IRRID):**

RR2-10.1136/bmjopen-2018-027442

## Introduction

Respiratory system maturation occurs in late gestation in preparation for the time of birth [1], extending into early childhood [2]. Essential for normal lung development, epigenetic mechanisms are influenced by the environment throughout gestation and postnatally [3]. Therefore, whether term or preterm, newborns might have immature ventilatory function that may foster respiratory instability [1].

Difficulties in assessing lung maturity arise when a single parameter is considered since the systems and organs may be at different stages of matureness. This is noticeable when judging maturity by gestational age when divergent pulmonary functional maturity is found between peers of the same age [4]. At different stages of development, organs can be affected by growth and differentiation factors released from other organs, as suggested by studies with multidimensional scaling and hierarchical cluster analysis of growth patterns during the fetal period [5].

Regarding the ability to interact with the external environment through the skin, a full-term newborn presents a complete or near entirely competent epidermal barrier at birth. The skin barrier at maturity, with an efficient stratum corneum achieved at around 34 weeks [6], prevents transepidermal water loss from the skin surface, maintaining the newborn’s temperature [7]. An immature skin barrier at birth leads to hypothermia, which in turn increases the likelihood of developing respiratory distress syndrome (RDS), intraventricular hemorrhage, late-onset sepsis, and mortality [8].

Similar to the skin, the lung shows signs of readiness for extrauterine life in the last trimester of gestation [9], with peak of alveoli maturation and surfactant production at 35 weeks of gestation [10]. Reduced surfactant, the major cause of RDS, causes low functional residual lung capacity, increasing the work of breathing and leading to terminal airway collapse. As a result, an increased ventilation-perfusion mismatch can lead to the need for ventilatory support [11], which in turn increases the metabolic and caloric demand to maintain temperature [12]. Although the point of view of care for lung immaturity is often related to the inability of the epidermal barrier to retain heat, there are few studies in this regard. Taesch et al (1972) [13] demonstrated that skin age is an indicator of lung age by studying rabbits [13].

In terms of access to lung maturity assessment, as well as advanced neonatal care, there is inequality around the world [14]. In these circumstances, knowing the risks of respiratory morbidity with accuracy might help in making more balanced decisions and determining the most appropriate care. Still, testing strategies based on gestational age for predicting the likelihood of RDS do not provide a complete approach to addressing the dilemma of indeterminate outcomes [10]. In response, noninvasive assessment methods have been proposed. A new photobiological device proved to correctly classify preterm newborns with 91.4% accuracy using a mathematical algorithm based on skin maturity and clinical adjusters [15]. In this context, the ability to accurately assess skin maturation and the potential synchrony of skin-lung development enables the study of a possible marker of lung maturation. The aim of this study was to assess the relationship between newborn skin maturity and RDS.

## Methods

### Setting

This study was carried out in 5 Brazilian urban reference centers for highly complex perinatal care in different regions: in the southeast, Hospital de Clínicas of Universidade Federal de Minas Gerais (as coordinator) and Hospital Sofia Feldman; in the south, Hospital of Universidade Luterana do Brasil; in the center-west, the Hospital Materno Infantil de Brasília; and in the northeast, the University Hospital of Universidade Federal do Maranhão.

### Ethics Approval

The trial protocol received approval from the independent ethics review board at each reference center under the number 81347817.6.1001.5149 at the Brazilian National Research Council. The procedures followed the Helsinki Declaration of 1975, as revised in 2013 [16], and all parents provided informed consent on behalf of their newborns before participating in the clinical trial.

### Study Design

This was a case-control nested in a prospective cohort study to investigate a secondary outcome within a single-blinded multicenter clinical trial investigation with a single group and single arm. The clinical trial protocol was disclosed in the World Health Organization’s International Clinical Trial Platform—Brazilian Clinical Trials (registered under trial number RBR-3f5bm5).

### Participants

In the primary cohort, a concurrent and sequential process enrolled newborns who were up to 24 hours old, had a gestational age of at least 24 weeks as determined by standard ultrasound, and were recruited between January 2, 2019, and May 30, 2021. Skin maturity assessment was conducted within the first 24 hours of life, regardless of the newborn’s location, whether it was in an incubator, heated crib, bassinet in the hospital room, or on the mother’s lap. All participants were followed for a period of 72 hours or until discharge or death, whichever occurred first, for the assessment of lung maturity. The examiner, who was blind to the results of the skin assessment, collected respiratory outcome data from medical charts. This study focused on the 72-hour follow-up data. More details about the study protocol can be found in a previous publication [17]. The timeframe of enrolment, intervention with the optical device, and respiratory outcome measurements is depicted in Table 1, adapted from Reis et al [18].

**Table 1:** Study timeline.

		Study period			
		Enrolment	Assessment	Close-out	Allocation
Time point		0 hours	0 hours	72 hours	Analysis
**Enrolment**					
	Eligibility	X			
	Informed consent	X			
Optical device intervention			X		
**Assessment and analysis**					
	Optical device data acquisition		X		X
	Standard ultrasound	X			X
	Case-control nested study		X	X	

We included newborns diagnosed with RDS based on clinical and radiological criteria after reviewing their clinical records. Newborns with immature lungs diagnosed with RDS formed the case group, and newborns with mature lungs without a respiratory diagnosis were randomly paired by gestational age ranges to form the control group. Newborns with extrapulmonary conditions, tachypnea due to causes other than prematurity, and diagnosis of infection were excluded.

### Skin Assessment

**Figure 1:**
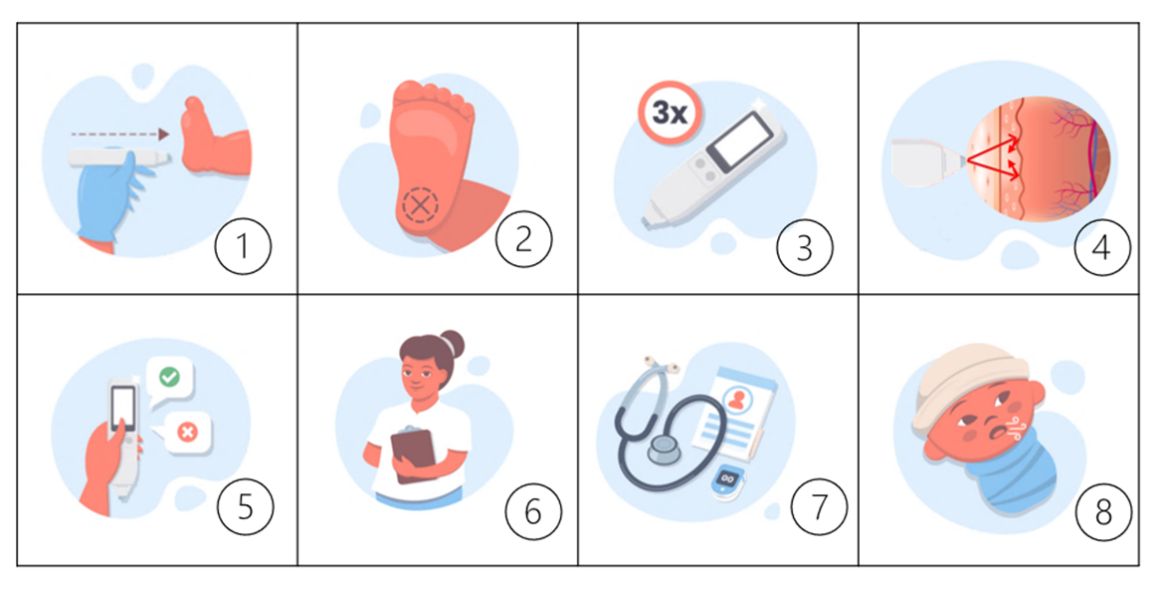
Steps of skin assessment in 8 steps: (1) the device touches the skin; (2) the standard body position for assessing skin reflectance in newborns is the sole; (3) 3 measurements are taken simultaneously; (4) the LED light interacts with the skin, scattering the light, and the returned light (reflectance) toward the sensor is processed by a control unit and stored for analysis; (5) the user inputs clinical data, such as birth weight and prenatal corticosteroid use; (6) the user collects vital data during the procedure; (7) the data are recorded and stored for analysis; and (8) the data processor estimates lung maturity using machine learning algorithms, associating light reflection and respiratory outcomes.

The skin assessment occurred with an optical device previously detailed [15] (Figure 1). Briefly, to obtain the skin reflectance, an LED sensor of wavelengths 400 to 1200 nm was touched to the newborn's sole for a few seconds to trigger 10 automated measurements. We performed 3 measurements, resulting in 30 automatic values to obtain the average reflection. The processor then captured the variations resulting from the interaction of the skin and LED light and kept it in storage for analysis. The data processor estimated lung maturity using machine learning algorithms. The evaluation advocated minimal manipulation, being performed in the position where the newborn was, after hand hygiene and sensor disinfection. The best body position to assess skin reflectance and possible influences, such as humidity, temperature, ambient light, and the skin tone of the newborn, were evaluated beforehand [19,20]. The reliability of skin assessment with the device was previously reported. The intraobserver and interobserver variability were 1.97% (95% CI 1.84%-2.11%) and 2.6% (95% CI 2.1%-3.1%), respectively [15].

The results obtained by the equipment were concealed from the researchers. The readings were stored in the processor and later transmitted to an electronic database, where they were stored on data servers. The results, apart from being inaccessible to the examiner, were also not shared with the professionals responsible for the child’s care in the actual scenario. This approach ensured that the study did not interfere with the clinical decisions made by the health care professionals.

### Clinical Data

To ensure proper data acquisition, all examiners were trained according to good clinical practice as recommended by the Brazilian Regulatory Health Agency [17]. Data related to respiratory outcomes were collected from medical records. During data curation, the senior clinician analyzed and confirmed the RDS diagnosis according to the guidelines previously described in the study protocol [21]. The framework of the clinical variables and skin acquisitions is available in Multimedia Appendix 1, as documented in the previous report by Reis et al [15].

We developed dedicated software to collect structured clinical data and associate them with the skin reflection of each newborn from 5 perinatal centers simultaneously. Examiners used individual sets of instruments, including a tablet, optical device, and paper versions of the clinical data forms. A double approach, on paper and electronically, allowed verification of clinical data for reliability and validity and was later validated by specialists in data curation.

### Primary Outcome

The primary outcome was the association between RDS occurrence and skin light reflection.

The diagnosis of RDS was based on a previously published clinical trial protocol [21]. In brief, it considered clinical, laboratory, and radiological findings. The observations made during the first 72 hours of life included tachydyspnea, the need for oxygen or ventilatory support after 24 hours of age, the requirement for surfactant replacement, and abnormal X-ray findings. The main radiological signs included underinflated lungs and a pattern of diffuse “ground glass” reticulogranular opacities, along with reduced lung volume and air bronchograms.

### Secondary Outcomes

The secondary outcomes were the association between the skin reflectance and NICU admission and the need for ventilatory support. Both invasive and noninvasive ventilatory supports were taken into account, including supplemental oxygen by nasal cannula or hood, nasal continuous positive airway pressure, noninvasive ventilation with biphasic positive airway pressure, and invasive mechanical ventilation through the endotracheal tube.

### Statistical Analysis

Descriptive statistics were used to explore the demographic and clinical characteristics of newborns according to groups of interest among the RDS and control groups. The analysis was performed by calculating the frequencies and percentages of categorical variables. The central tendency, mean (SD), median (IQR), and dispersion were calculated for quantitative variables. The independent sample *t* test or Mann-Whitney test was used to compare continuous variables, and the *χ*^2^ test or Fisher exact test was used to compare categorical variables according to the nature of their distribution.

The inferential statistical analysis evaluated the relationship between skin reflectance and outcome occurrence. The sensor acquisition produced by the skin reflection was the independent variable. Logistic regression was used to identify potential influencers of RDS occurrence as birth weight and antenatal corticoid exposition. Similar analyses were conducted with the secondary outcomes, NICU admission and ventilatory support needs. Inference was estimated by calculating odds ratios (ORs) with 95% CIs. The Nagelkerke *R*^2^ was used to measure how well the independent variables explained the variance in the model. A Wald test was used to confirm if a set of independent variables were collectively significant for the model. The variables corresponding to *P* values <.05 in the univariate analysis were selected for the multivariate analysis, and analyses were performed using the available data with imputation of the missing data. The statistical software SPSS 25.0 (IBM) was used for the analysis.

## Results

**Figure 2:**
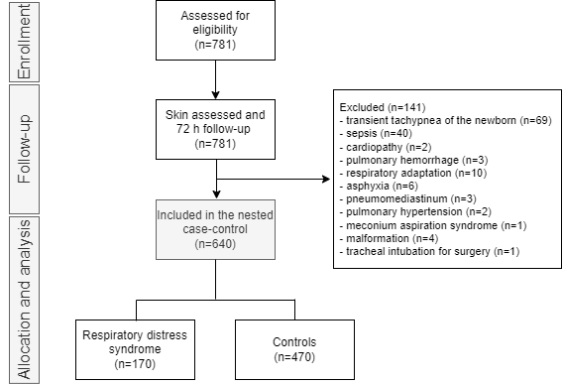
Flow diagram of participants included in the 72-hour follow-up study.

At the end of the cohort study of 781 newborns, 640 were selected for the case-control analysis according to the eligibility criteria (Figure 2).

The main characteristics of antenatal care and newborn infants are shown in Table 2. There were 5 missing data points from 4 newborns, including information on the use of antenatal corticosteroid therapy for fetal maturation exposition (ACTMF), the presence of diabetes, the first minute Apgar score, and the fifth minute Apgar score. According to comparisons between groups, newborns with RDS had a younger gestational age (31.6 vs 39.1 weeks, *P<*.001) and lower birth weight (1491 vs 3121 grams, *P<*.001) than controls. During the 72-hour follow-up, significantly different rates were found between groups regarding NICU admission (RDS: n=170, 100%; control: n=16, 3.4%; *P<*.001), mortality (RDS: n=5, 9.2%; control: n=0, 0%; *P<*.001), and the need for ventilatory support (RDS: n=160, 100%; control: n=6, 1.3%; *P<*.001).

**Table 2:** Clinical characteristics of the studied newborns.

Variable			Total (n=640)	RDS^a^ (n=170)	Controls (n=470)	*P* value (RDS vs control)
**Maternal characteristics, n (%)**						
	ACTMF^b^		192 (30)	147 (87)	45 (9.6)	<.001
	Diabetes		90 (14.1)	41 (24.1)	49 (10.4)	<.001
	HDP^c^		129 (20.2)	66 (38.8)	63 (13.4)	<.001
	Multiple gestation		101 (15.8)	67 (39.4)	34 (7.2)	<.001
**Demographic data at birth**						
	GA^d^ (weeks), median (IQR)		38.1 (33.8-39.9)	31.6 (29.9-33.4)	39.1 (37.4-40.1)	<.001
	Preterm, n (%)		250 (39.1)	169 (99.4)	81 (17.2)	<.001
	Sex, male, n (%)		320 (50)	89 (52.4)	231 (49.1)	.53
	Birth weight (g), median (IQR)		2688 (905)	1491 (513)	3121 (561)	<.001
	**Birth weight classification, n (%)**					<.001
		>2500 g	420 (65.6)	4 (1)	416 (99)	
		LBW^e^	130 (20.3)	79 (46.5)	51 (10.9)	
		VLBW^f^	58 (9.1)	55 (32.4)	3 (0.6)	
		ELBW^g^	32 (5)	32 (18.8)	0 (0)	
	1-minute Apgar score, median (IQR)		9 (8-9)	8 (6-9)	9 (8-9)	<.001
	5-minute Apgar score, median (IQR)		9 (9-10)	9 (8-10)	9 (9-10)	<.001
	Neonatal resuscitation first steps, n (%)		268 (41.9)	160 (94.1)	108 (23)	<.001
	Neonatal resuscitation steps, PPV^i^, n (%)		95 (14.8)	75 (44.1)	20 (4.3)	<.001
	Neonatal resuscitation steps, intubation, n (%)		26 (4.1)	24 (14.1)	2 (0.4)	<.001
	Advanced resuscitation, n (%)		2 (0.3)	2 (1.2)	0 (0)	<.001
**Follow-up within 72 hours, n (%)**						
	NICU^j^ admission		186 (29.1)	170 (100)	16 (3.4)	<.001
	Discharge		395 (61.7)	0 (0)	395 (84)	<.001
	Mortality		5 (0.8)	5 (2.9)	0 (0)	.07
	Incubator		166 (25.9)	152 (89.4)	14 (3)	<.001
	**Ventilatory support**		176 (27.5)	170 (100)	6 (1.3)	<.001
		MV^k^	54 (8.4)	51 (30)	3 (0.6)	<.001
		NIV^l^	43 (6.7)	43 (25.3)	0 (0)	<.001
		CPAP^m^	156 (24.4)	153 (90)	3 (0.6)	<.001
		NC^n^	2 (0.3)	2 (1.2)	0 (0)	<.001
	Surfactant therapy		73 (11.4)	73 (42.9)	0 (0)	<.001

^a^RDS: respiratory distress syndrome.

^b^ACTMF: antenatal corticosteroid therapy for fetal maturation exposition.

^c^HDP: hypertensive disorders of pregnancy.

^d^GA: gestational age.

^e^LBW: low birth weight (<2500 g).

^f^VLBW: very low birth weight (<1500 g).

^g^ELBW: extremely low birth weight (<1000 g).

^h^Not available.

^i^PPV: positive pressure ventilation.

^j^NICU: neonatal intensive care unit.

^k^MV: invasive mechanical ventilation.

^l^NIV: noninvasive mechanical ventilation with bilevel-positive airway pressure.

^m^CPAP: continuous positive airway pressure.

^n^NC: oxygen by nasal cannula.

**Figure 3:**
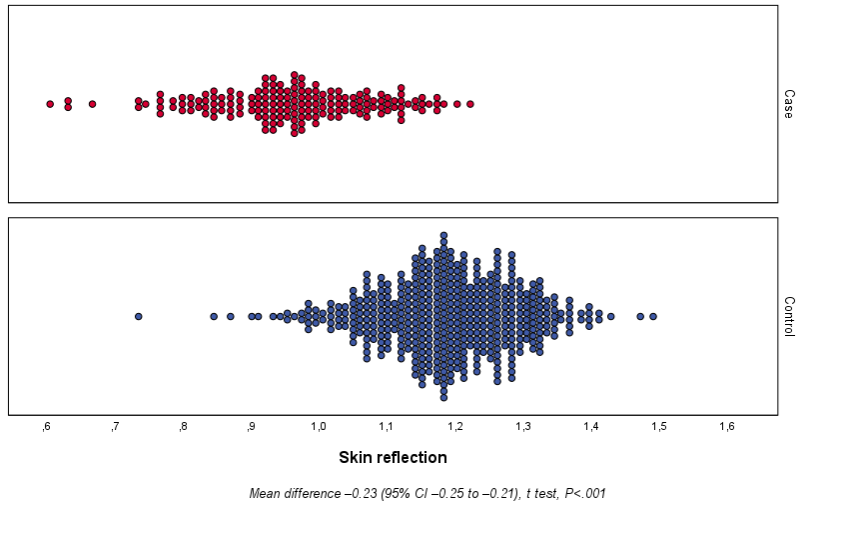
Primary outcome: newborn skin reflection acquired on the sole of the foot on the first day of life for the case and control groups.

Concerning the primary outcome, different reflectance of the skin over the sole was observed between the groups studied (Figure 3). The reflectance range for the RDS group was 0.588-1.208 with a mean of 0.945 (SD 0.118), and that for the control group was 0.717-1.274 with a mean of 1.172 (SD 0.103). The mean difference of reflectance between groups was –0.227 (95% CI –0.246 to –0.208; *P<*.001).

The univariate analysis showed a correlation between skin reflection and RDS. The skin reflectance was associated with RDS in the univariate analysis (OR 0.982, 95% CI 0.979-0.985, *R*^2^=0.632, *P<*.001) as well as in the cofactor-adjusted analysis (OR 0.994, 95% CI 0.990-0.998, *R*^2^=0.843, *P<*.001) (Table 3). Skin reflection was associated with RDS regardless of the inclusion of ACTMF and birth weight in the multivariate model.

**Table 3:** Univariate and multivariate analyses of the association between skin maturity and the occurrence of respiratory distress syndrome acquired by the optical device.

Variable	Univariate analysis			Multivariate analysis		
	OR^a^ (95% CI)	*P* value (Wald test)	R^2^	OR (95% CI)	*P* value (Wald test)	R^2^
Skin reflection^b^	0.982 (0.979-0.985)	*<*.001	0.632	0.994 (0.990-0.998)	.001	0.843
Birth weight	0.995 (0.994-0.996)	*<*.001	0.825	0.996 (0.996-0.997)	*<*.001	
ACTMF^c^	63.106 (36.655-108.646)	*<*.001	0.621	2.854 (1.207-6.749)	.02	

^a^OR: odds ratio.

^b^OR and 95% CI values for skin reflection are × 10^3^.

^c^ACTMF: antenatal corticosteroid therapy for fetal maturation exposition.

**Figure 4:**
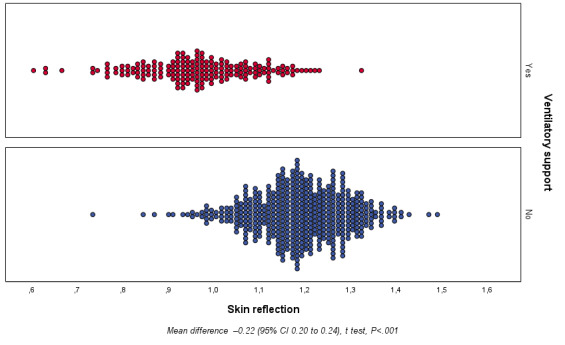
Secondary outcome: newborn skin reflection acquired on the sole of the foot in the first 24 hours of life according to ventilatory support use (yes or no).

**Figure 5:**
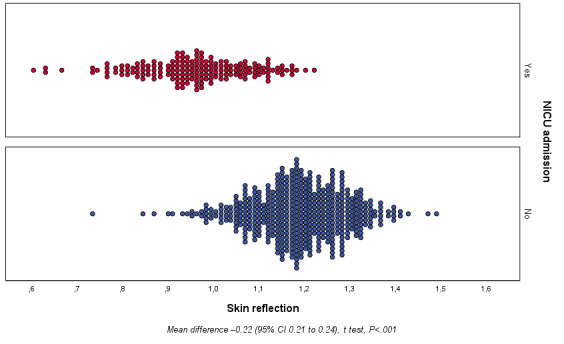
Secondary outcome: newborn skin reflection acquired on the sole of the foot in the first 24 hours of life according to NICU admission (yes or no). NICU: neonatal intensive care unit.

Secondary outcome data showed differences in skin reflectance between the studied groups for both ventilatory support use (yes vs no; Figure 4) and NICU admission (yes vs no; Figure 5). Regarding ventilatory support, the skin reflectance ranged from 0.588 to 1.305 with a mean of 0.952 (SD 0.009) and 0.717 to 1.474 with a mean of 1.172 (SD 0.005), for the yes and no groups, respectively. The mean difference was 0.219 (95% CI 0.200-0.238; *P<*.001). For NICU admission, the skin reflectance ranged from 0.588 to 1.304 with a mean of 0.953 (SD 0.009) and 0.717 to 1.473 with a mean of 1.176 (SD 0.005) for the yes and no groups, respectively. The mean difference was 0.223 (95% CI 0.205-0.241; *P<*.001).

The univariate and multivariate analyses of secondary outcomes are summarized in Tables 4 and 5 for ventilatory support use and NICU admission, respectively. Skin reflection was associated with the need for ventilatory support in the univariate analysis (OR 0.983, 95% CI 0.981-0.986, *R*^2^=0.598, *P<*.001) as well as in the cofactor-adjusted analysis (OR 0.996, 95% CI 0.992-0.999, *R*^2^=0.814, *P*=.01). Similarly, there was an association between skin maturity and NICU admission in the univariate analysis (OR 0.928, 95% CI 0.979-0.985, *R*^2^=0.635, *P<*.001) and multivariate analysis (OR 0.994, 95% CI 0.990-0.998, *R*^2^=0.867, *P*=.004).

**Table 4:** Univariate and multivariate analyses of the association between skin maturity and the need for ventilatory support during the first 72 hours of life.

Variable	Univariate analysis			Multivariate analysis		
	OR^a^ (95% CI)	*P* value (Wald test)	R^2^	OR (95% CI)	*P* value (Wald test)	R^2^
Skin reflection^b^	0.983 (0.981-0.986)	*<*.001	0.598	0.996 (0.992-0.999)	.01	0.814
Birth weight	0.996 (0.995-0.993)	*<*.001	0.801	0.997 (0.996-0.998)	*<*.001	
ACTMF^c^	56.108 (33.307-94.520)	*<*.001	0.607	2.677 (1.209-5.924)	.01	

^a^OR: odds ratio.

^b^OR and 95% CI values for skin reflection are × 10^3^.

^c^ACTMF: antenatal corticosteroid therapy for fetal maturation exposition.

**Table 5:** Univariate and multivariate analyses of the association between skin maturity and neonatal intensive care unit admission during the first 72 hours of life.

Variable	Univariate analysis			Multivariate analysis		
	OR^a^ (95% CI)	*P* value (Wald test)	R^2^	OR (95% CI)	*P* value (Wald test)	R^2^
Skin reflection^b^	0.928 (0.979-0.985)	*<*.001	0.635	0.994 (0.990-0.998)	.004	0.867
Birth weight	0.995 (0.994-0.996)	*<*.001	0.852	0.996 (0.995-0.997)	*<*.001	
ACTMF^c^	72.288 (42.238-123.715)	*<*.001	0.648	2.908 (1.223-6.9155)	.02	

^a^OR: odds ratio.

^b^OR and 95% CI values for skin reflection are × 10^3^.

^c^ACTMF: antenatal corticosteroid therapy for fetal maturation exposition.

## Discussion

### Principal Findings

The main aim of this study was to demonstrate the use of skin maturity assessment as a potential marker of lung maturation. We found an association between skin immaturity and the occurrence of RDS, as well as a similar association with other respiratory outcomes, such as NICU admission and the need for ventilatory support. Skin reflectance at newborns’ soles, assessed within the first 24 hours of life using an optical device, indicated its maturity. Respiratory outcomes related to lung maturity at 72 hours of life were obtained from medical records. These results could enhance neonatal care, as knowledge of lung maturity, regardless of the newborn’s gestational age, can facilitate individualized care in the first hours of life.

Our findings reinforce the theory of the parallel development of the organs, highlighting the similarity between stages, which may allow the indirect evaluation of an organic system based on the measurements of another, regardless of age [13]. Studies in animal models have shown the similarity in the process of lipid production between the stratum corneum and pulmonary surfactant [22]. Predicting surfactant deficiency before respiratory deterioration depends on a combination of clinical signs and lung imaging [23]. Therefore, a timely indication of surfactant therapy may be postponed due to various factors, such as the lack of specificity in the initial phase of the imaging methods, the absence of the exam in low- and middle-income countries, and the masked signs of RDS severity by early continuous positive airway pressure protocols [24,25]. In this context, skin assessment appears to be a potential alternative, as this study demonstrated an association between skin reflectance and the need for ventilatory support in the first 72 hours of life. To the best of our knowledge, this is a pioneering study of pulmonary assessment using an indirect and noninvasive method.

This study presented no intention of predicting RDS or other complications related to pulmonary immaturity since it had a nested case-control design. Removing confounding diseases, such as sepsis, malformations, and other respiratory diagnoses, was essential to analyze the relationship between skin maturity and RDS. This analysis plan was stated in the clinical trial protocol [18]. Additionally, ACTMF and birth weight were included in the multivariate model to investigate the independent and adjusted association of skin-lung maturity. A comprehensive sample of newborns with train-test procedures on machine learning approaches is still necessary to provide valuable models of RDS prediction using skin reflection.

Furthermore, this study found an association between skin reflection and NICU admission when adjusted by the cofactors of birth weight and ACTMF. Previous reports demonstrated the likelihood of preterm newborns, especially those with low birth weight, requiring NICU monitoring due to hypothermia [22]. This population is prone to uncontrolled heat loss, leading to hypoglycemia and hypoxemia, resulting in metabolic acidosis and associated respiratory distress [26]. Predicting which infants will become symptomatic of RDS is not always possible before birth. If not recognized and managed quickly, respiratory distress can escalate to respiratory failure and cardiopulmonary arrest [27]. Clinical evaluation of fetal lung maturity based on the analysis of the lecithin to sphingomyelin ratio and lamellar body count demands amniocentesis, which is an invasive procedure that poses potential risks, such as preterm labor, fetomaternal hemorrhage, or even death [28]. Furthermore, there is a large difference in sensitivity and specificity among laboratory analyses depending on the test [25]. Therefore, antenatal assessment of fetal lung maturity is limited, and we believe that a noninvasive method to assess lung maturity at birth can meet the actual clinical needs.

Medical technologies for monitoring fetal and maternal health are not equally accessible [29]. In low- and middle-income countries, among the challenges is the transfer of the newborn to specialized services due to suboptimal modes of transport and difficult and time-consuming routes [30]. Therefore, early assessment of lung maturity could improve resource allocation, supporting the indication for transport, and potentially lowering the mortality risk. The studied device can be easily applied by several health professionals, favoring assistance especially in low-resource settings. This study is the first step in this context, showing a possible agreement between lung and skin maturation, relying on RDS strictly due to immaturity.

### Limitations

To assess pulmonary readiness for the extrauterine life, our data analysis was based on the RDS scenario strictly caused by immaturity and is therefore unable to predict the occurrence of RDS. Since this case-control study excluded other neonatal conditions, a comprehensive analysis including all causes of respiratory distress at birth is necessary.

### Strengths

As far as we know, this was the first study to demonstrate the phenomenon of association between skin and lung maturity assessed postnatally. Through an accessible technological tool that can be integrated into current clinical practice, a therapeutic possibility arises for the indirect assessment of lung maturity.

Despite the case-control methodology, data collection was prospective, the research protocol was previously published, and the team was trained and certified [17].

### Future Directions

To develop and validate a predictive model for RDS, a study that includes all causes of respiratory distress at birth is necessary. We already have a study underway for this purpose.

### Conclusion

This study showed the potential for identifying RDS and immediate respiratory complications in the first 72 hours of life through skin assessment based on the synchronous development of the lungs and skin. The results, however, may not be applicable for predicting RDS, ventilatory support use, or NICU admission.

## Appendix

Multimedia Appendix 1 Database of clinical variables collected from each newborn.

## Abbreviations

ACTFM: antenatal corticosteroid therapy for fetal maturation
NICU: neonatal intensive care unit
RDS: respiratory distress syndrome

## Data Availability

The data sets generated analyzed in this study are available from the corresponding author upon reasonable request. Access to the data is provided after anonymization to ensure ethical and legal sharing while preserving the confidentiality of the study participants.
